# Relationships between executive function, mentalization and earthquake-related stress reactions in adults

**DOI:** 10.1186/s40359-025-03349-w

**Published:** 2025-09-26

**Authors:** Feyruz Usluoglu

**Affiliations:** Psychology Department, Toros University, Bahçelievler District, Mersin, Turkey

**Keywords:** Mentalizing, Executive functioning, Post-traumatic stress, Earthquake

## Abstract

**Background:**

Academic research shows that mentalizing (MZ) and executive function (EF) are important components in maintaining mental health and in responding adaptively to stressors. Therefore, they may be better associated with post-traumatic stress-related mental health problems. However, studies of the relationship between EF, MZ and post-traumatic stress (PTS) symptoms have mostly focused on childhood traumas and related mental health problems. Few studies have examined the relationship of these variables with trauma types common in adulthood, such as exposure to an earthquake.

**Objective:**

This study investigated the relationship between EF, MZ, and PTS symptoms, and tested whether specific dimensions of MZ moderate the EF–PTS link in a sample of adults exposed to the February 2023 earthquakes in Turkey.

**Method:**

I collected data from the three provinces in Turkey that experienced the most destruction and loss of life due to earthquakes that occurred on February 6 and 20, 2023. The participants completed the Adulthood Executive Functioning Inventory (ADEXI/ EF), the Certainty About Mental States Questionnaire (CAMSQ/MZ), and the Post-Traumatic Stress Disorder Checklist (PCL). Accordingly, I tested the moderating effect of CAMSQ self-certainty (SC), other-certainty (OC), and hypermentalizing (HMZ, i.e., other–self discrepancy) scores on the relationship between EF and PCL scores.

**Results:**

The results indicate that SC (*B* = − 0.007, 95% CI [− 0.024, 0.011], *p* = .462) and OC (*B* = − 0.009, 95% CI [− 0.028, 0.009], *p* = .309) had no moderating effect on the relationship between EF and PCL, whereas HMZ showed a significant moderating effect (*B* = − 0.395, 95% CI [− 0.740, − 0.049], *p* = .025). These findings suggest that both EF impairment (*B* = 1.770, 95% CI [1.078, 2.462], *p* < .001) and HMZ (*B* = 15.922, 95% CI [4.426, 27.419], *p* = .007) are strongly associated with PTS reactions among adults who have experienced earthquake trauma and challenging post-earthquake conditions.

**Conclusion:**

This study highlights the relationship between two important neurocognitive components and post-traumatic stress symptoms in adults. The results suggest that higher EF and optimal mentalizing may be related to better coping with prolonged post-disaster challenges. Interventions such as targeted cognitive training and mentalization-based strategies could be explored in future research to examine their potential for reducing vulnerability to chronic stress reactions.

## Highlight

I examined the associations between executive functions, mentalization, and post-traumatic stress symptoms in adult earthquake survivors.

Executive functions and mentalizing may be important components in maintaining mental health and demonstrating adaptive responses to the effects of stress.

Our research results suggest that executive dysfunction and too much of others’ mental states (hypermentalizing) are significantly associated with posttraumatic stress reactions in adults who experienced earthquake trauma and challenging post-earthquake conditions.

## Introduction

In February 2023, Turkey was struck by a series of devastating earthquakes whose scale was unprecedented, even for this earthquake-prone region. The initial earthquakes, which registered magnitudes of 7.7 and 7.6, were centered in Kahramanmaraş and occurred on February 6, affecting 11 provinces and resulting in 50,783 deaths and 115,353 injuries, according to official data [1]. Subsequently, on February 20, an earthquake with a magnitude of 6.4 struck Hatay [1]. These earthquakes caused the collapse of, or severe damage to, over 306,467 buildings as of January 22, 2024, leading to significant material losses[2] The destruction displaced 3.3 million people, with nearly 2 million forced to live in temporary accommodations, such as tent camps and container settlements.

In response to this disaster, at a conference held in March 2023, the Turkish government, in partnership with the United Nations Development Programme, the World Bank, and the European Union, conducted a preliminary post-disaster needs assessment to inform reconstruction and encourage donor contributions. The earthquakes, which devastated a region spanning 110,000 square kilometers, led to severe human and economic losses and had significant social and psychological consequences. Survivors have been observed to experience various psychological problems, including post-traumatic stress disorder (PTSD) symptoms, due to the multidimensional impacts of the [3]. In addition to the onset or intensification of psychiatric disorders, such as PTSD, depression, anxiety, substance abuse, grief reactions, and sleep disturbances, it is well known that natural disasters frequently cause psychological distress among exposed populations [4, 5]. Given the high prevalence of post-earthquake PTSD, identifying the variables that can increase the risk of this condition is essential. This emphasizes the necessity for more research in this area.

Exposure to traumatic events associated with earthquakes has been demonstrated to be crucial in increasing the likelihood of developing PTSD [6]. Furthermore, it is crucial to recognize that prolonged or intense stress has been associated with impairments in both executive function (EF) and mentalizing (MZ). This is a pressing issue that needs to be addressed, as dysregulation of EF and MZ has been linked to lower quality of life in otherwise healthy individuals [7].

Executive functions, which primarily rely on the prefrontal cortex and the reticular–thalamic system [8], are commonly described as higher-order cognitive processes that are necessary for goal-directed behaviors. These processes include working memory, cognitive flexibility, and inhibition [9, 10]. Cognitive flexibility is seen as a third element, while most current research focuses on working memory and inhibitory aspects [9, 11]. Working memory is involved in the processes of executing complex cognitive tasks by storing and managing verbal and nonverbal information. Inhibitory control, on the other hand, refers to the ability to control attention, thoughts, and emotions [12].

Mentalizing refers to the capacity to understand and interpret oneself and others'mental states [13]. MZ has been associated with emotional regulation [14], and may contribute to psychological resilience by helping people cope with psychological symptoms [15].

EF skills are associated with the ability to adapt flexibly to changing conditions and challenges [10]. Similarly, mentalization involves developing self-regulation skills by certaining one's own mental states and adapting to complex social situations by certaining the mental states of others. However, there is a relationship between intense and prolonged stress and EF and MZ disorders [7, 13]. For example, both cognitive abilities show significant impairment in individuals with PTSD [16, 17].

Traumatic stress and arousal can impair mature and functional MZ abilities of adult individuals [16, 17]. Especially in stressful situations, evaluating ones’s own situation and feelings as the only possible reality can reduce the possibility of assuming alternative perspectives [18]. Thus, MZ impairments may affect individuals’ ability to perceive and benefit from the social support offered to them. In addition, when individuals with PTSD are exposed to reminders of trauma, they may develop anxiety because they do not make appropriate assessments [19]. Similarly, due to the role of EF deficits have been associated with difficulties processing and regulating emotional states, individuals with EF impairments may develop PTS symptoms [20]. In this respect, EF and MZ impairments may have an important relationship with the development and maintenance of stress reactions.

### The current study

EF [21, 22] and MZ [23, 24] are important components in the maintenance of mental health and adaptive responses to stressors. However, research has yielded different results regarding the casual relationship between these components [25, 26]. However, it has been stated that impairments in EF can cause impairments in MZ functions [27]. Moreover, the relationship of both EF and MZ with PTS is mostly examined in the context of childhood trauma. A limited number of studies have examined these variables, doing so both separately and in the context of trauma exposure in adulthood [28–30]. In this context, it has been suggested that the relationship between executive function and PTSD be further investigated in the context of different types of traumas [16]. In addition, the tools used to assess MZ have limitations such as providing a one-dimensional assessment [19, 31, 32] and failing to address the difference between understanding oneself and others'mental states [32]. So, it has been specifically recommended that researchers examine the different components of MZ [19].

Like the work on the relationship between EF and MZ, the relationship between EF, MZ, and PTS symptoms has limitations, in adulthood and in the context of different types of traumas (e.g., earthquake trauma). Considering these limitations, this study aimed to examine the relationship between EF, MZ, and trauma stress reactions in a sample of adults who had been exposed to an earthquake. To this end, I hypothesized that self-certainty, other-certainty, and hypermentalizing (HMZ, also referred to as other-self-discrepancy) [32] might moderate the relationship between executive functions [33] and post-traumatic stress reactions [34]. We believe that the findings of this research will contribute to the existing literature on the relationship between these variables and may inform future work on clinical interventions for earthquake trauma.

## Method

### Participants

Participants were eligible for inclusion if they had been affected by the 6 February 2023 earthquakes, were between 18 and 65 years of age, could understand and speak Turkish, and had no mental health conditions that would hinder their ability to cooperate. Individuals who did not meet these criteria were not included in the data collection process. Therefore, no participants were excluded at the analysis stage. As a result, I recruited 575 adults aged 18–65 years (mean = 34.10, standard deviation [SD] = 11.52). The data were collected by convenience sampling from individuals in Adıyaman (18.3%; OSM 1 K-14, İpekli Şehir Hastanesi K2B) Kahramanmaraş (29.9%; Havaalanı Container City, Necip Fazıl Container City), and especially Hatay (52.0%; Katar 4 Container City, Turunçlu Container City, İbrahim Çeçen Container City), which were the cities most affected by the earthquake centered in Kahramanmaraş, Turkey on February 6 and February 20, 2023. A total of 452 participants (78.6%) lived in container cities throughout the study. Of the participants, 371 (64.5%) were female, and 204 (35.5%) were male. In terms of educational background, most of the participants had an undergraduate degree (*n* = 250, 43.5%). Of the participants, 259 (45.0%) were single, and 301 (52.3%) were married. The participants were asked six questions about their experiences with being trapped under rubble resulting from the earthquake as well as their degrees of kinship with victims who died in the earthquake (Table 1).

**Table 1:** Participant responses regarding exposure to earthquakes

Questions	Response	*n*	%
1. Were you trapped under rubble?	Yes	159	27.7
	No	416	72.3
2. Have you suffered losses among your first-degree relatives (parents, spouse, children)?	Yes	145	25.2
	No	430	74.8
3. Have you lost any second-degree relatives (grandparents, siblings, and grandchildren)?	Yes	132	23.0
	No	443	77.0
4. Have you lost any third-degree relatives (aunts, uncle, and nephews)?	Yes	158	27.5
	No	417	72.5
5. Have you lost any of your fourth-degree relatives (cousins)?	Yes	194	33.7
	No	381	66.3
6. Have you suffered the loss of other people you were close to (e.g., friends or neighbors)?	Yes	310	53.9
	No	265	46.1

### Procedure

I collected data for this study between March and August 2024 from 575 native Turkish participants. The participants were asked to complete a set of questionnaires within a survey. Before completing the questionnaires, the participants were provided with information regarding the study’s purpose, and written informed consent was obtained. Participants who completed the consent form, knew how to read and write in their native language, and volunteered to participate in the study were included in the data collection process. The researcher did not manipulate this process in any way, and the participants responded freely. They were not given any rewards or incentives for their participation. The study complied with the regulations stipulated by the university ethics committee (masked for review).

### Measurements

#### The adult EF inventory

The Adulthood Executive Functioning Inventory (ADEXI; [33] is a 14-item self-report questionnaire scored on a 5-point Likert scale (1 = strongly disagree, 5 = strongly agree) in two dimensions (working memory and inhibition). Higher scores indicate a decline in EF. In the original study, the Cronbach alpha scores were 0.89, 0.88, and 0.72 for the dimensions of total score, working memory, and inhibition, respectively. The scale was adapted to Turkish by Alpay and Kaya-Kızılöz [35]. In this study, the Cronbach alpha scores were 0.80, 0.83, and 0.73 for working memory and inhibition, and total score of scale respectively. In the current study, I used the total score of the scale (α = 0.86).

#### The post-traumatic stress disorder checklist

In this research, I used the Post-Traumatic Stress Disorder Checklist 20 (PCL; [34, 36]) to examine the participants’ PTS symptoms. The PCL-5 is a 20-item self-report scale that assesses DSM-5 PTSD symptom severity. The items on the PCL-5 correspond to DSM-5 symptom criteria: Criterion B for intrusion (Items 1–5), Criterion C for avoidance (Items 6–7), Criterion D for negative changes in cognition and mood (Items 8–14), and Criterion E for changes in arousal and reactivity (Items 15–20). Symptom severity is rated from 0 (none) to 4 (extreme), and the scores are summed to yield a total severity score. The scale was translated into Turkish by Boysan et al., [37]. In the current study, the Cronbach alpha scores were calculated as 0.93.

The certainty about mental states questionnaire (CAMSQ)

In this study, I used the Certainty About Mental States Questionnaire (CAMSQ; 32) to assess the participants’ MZ skills. The CAMSQ assesses the capacity of the respondent to MZ about themselves (Self-Certanity, SC) and others (Other-Certanity, OC) in two dimensions of 10 items each. The CAMSQ is a 7-point Likert-type scale (never = 1, rarely = 2, sometimes = 3, half the time = 4, often = 5, usually = 6, always = 7) that assesses two maladaptive forms of MZ. One is indicated by low scores on the SC scale, and the other corresponds to the situation in which OC exceeds SC (yielding an other-self-certainty discrepancy score > 0). The first of these is called hypomentalizing and the second hypermentalizing (HMZ). The internal consistency coefficients are ω = 0.91/0.89 for the SC scale and ω = 0.90/0.88 for the OC scale. The CAMSQ was adapted into Turkish by [38] and in that study, the Cronbach’s α and McDonald’s omega (ω) values for the SC subdimension of the scale were 0.89 and 0.90, respectively. For the subdimension OC, these values were 0.88 and 0.89, respectively. In the current study, the Cronbach alpha scores for SC, OC, and the total scale were calculated as 0.82, 0.80, and 0.88, respectively.

### Data analysis

All analyses were conducted using JASP 0.18.1 [39] and R 4.2.2 [40].

### Statistical method & data cleaning

I handled missing data in R [40] with mice library [41]. I determined the minimum number of participants according to the recommendations of Bryman and Cramer [42]. I checked the normality assumptions in the data using the skewness and kurtosis scores, and all variables’ scores were within acceptable limits [43]; maximum skewness = −0., maximum kurtosis = 2.808). In a statistical analysis, I calculated the descriptive statistics and investigated the relationships between the study variables. I used a Pearson correlation analysis to assess the links between the variables. For this analysis, I used the JASP 0.18.1 program. To test our model, I used multiple moderation analysis. In this analysis, I tested moderator roles, self-mentalization, other-mentalization, and HMZ in the relationship between EF and traumatic stress. All indirect effects were tested through bootstrapping using 10,000 resamples and 95% bias-corrected confidence intervals (CIs). I employed R 4.2.2 to analyze multiple moderations with the lavaan library [44].

## Results

### Preliminary analyses

The means, standard deviations, and Pearson correlation coefficients among the study variables are presented in Table 2. The correlation analysis revealed several significant relationships among the variables. A significant negative correlation was found between EF and CAMSQ (*r* = − 0.317, *p* < 0.01), OC (*r* = − 0.169, *p* < 0.01), and SC (*r* = − 0.343, *p* < 0.01), indicating that higher levels of EF are associated with lower scores in these CAMSQ dimensions. Conversely, a positive correlation emerged between EF and PCL (*r* = 0.370, *p* < 0.01), suggesting that higher EF scores are associated with higher PCL scores. Additionally, a moderate positive correlation was identified between EF and HMZ (*r* = 0.203, *p* < 0.01). CAMSQ was significantly positively correlated with OC (r = 0.818, *p* < 0.01) and SC (*r* = 0.878, *p* < 0.01), underscoring the strong relationships between these constructs. Moreover, OC demonstrated significant positive correlations with SC (r = 0.637, *p* < 0.01) and PCL (*r* = 0.307, *p* < 0.01). However, SC was negatively correlated with HMZ (*r* = − 0.423, *p* < 0.01), further highlighting the complex interplay among these variables. PCL also had a significant positive correlation with HMZ (*r* = 0.201, *p* < 0.01). These findings provide a nuanced understanding of the relationships between EF, CAMSQ, PCL, and HMZ in the study sample.

**Table 2:** Descriptive statistics and correlations between study variables

Variable	1	2	3	4	5	6
1. EF	-					
2. CAMSQ	−0.317^**^	-				
3. SC	−0.343^**^	0.878^**^	-			
4. OC	−0.169^**^	0.818^**^	0.637^**^	-		
5. PCL	0.370^**^	0.1270^**^	0.079	0.307^**^	-	
6. HMZ	0.203^**^	−0.196^**^	−0.423^**^	0.155^**^	0.201^**^	1
*M*	31.994	108.092	52.085	54.906	44.412	-
*SD*	9.370	19.811	10.371	11.327	16.924	-

*EF* The Adult EF Inventory, *CAMSQ* The Certainty About Mental States Questionnaire, *SC* self- certainty, *OC* other-certainty, *PCL* The PTS Disorder Checklist, *HMZ* HMZ (other-self-discrepancy), ^**^p<.01

### Moderation results

I examined the moderating role of OC, SC, and HMZ in the indirect effect of EF on PCL. To investigate this relationship, the interaction between EF*OC interaction, EF*SC interaction, and EF*HMZ interaction was examined. The moderation analysis indicated that several variables were significantly related to PCL. EF showed a strong positive relationship with PCL (*B* = 1.770, 95% CI [1.078, 2.462], *p* < 0.001), suggesting that higher levels of EF are associated with increased PCL scores. OC was also significantly related to PCL (*B* = 0.865, 95% CI [0.289, 1.441], *p* = 0.003), indicating a positive association between this factor and PCL. Additionally, HMZ demonstrated a significant relationship with PCL (*B* = 15.922, 95% CI [4.426, 27.419], *p* = 0.007), with a substantial effect size, highlighting that higher levels of HMZ are associated with increased PCL symptoms. The interaction between EF and HMZ was significant (*B* = −0.395, 95% CI [−0.740, −0.049], *p* = 0.025), suggesting that higher levels of HMZ moderate the relationship between EF and PCL, potentially strengthening the association between EF and PCL scores. However, the interactions between EF and OC (*B* = −0.009, 95% CI [−0.028, 0.009], *p* = 0.309) and EF and SC (*B* = −0.007, 95% CI [−0.024, 0.011], *p* = 0.462) were not statistically significant. The overall model explained 29.8% of the variance in PCL scores (R^2^ = 0.298), indicating a moderate fit.

**Fig. 1:**
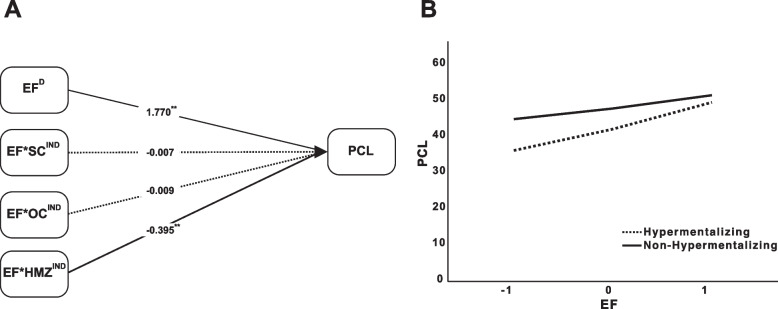
Moderation analysis results. **A** Path diagram and standardized coefficients from the moderation analysis. Solid lines indicate significant effects, and dashed lines indicate non-significant effects. Superscript D indicates direct effects, IND indicates interaction (moderated) effects. **B** Visualization of the moderating effect of hypermentalizing. The dashed line indicates the hypermentalizing group, and the solid line indicate the non-hypermentalizing group

The effect sizes for the interaction effects were calculated using the *f*^2^ statistic [45] to assess the moderating role of each interaction term in predicting PCL. The interaction between EF and HMZ demonstrated a small but meaningful effect size (*f*^2^ = 0.011), indicating that this interaction contributes to explaining the variance in PCL scores. In contrast, the interactions between EF and OC (*f*^2^ = 0.004) and between EF and SC (*f*^2^ = 0) had very small or negligible effect sizes, suggesting that these interactions do not significantly impact PCL scores. Overall, the interaction between EF and HMZ had the most notable influence, while the others showed minimal contributions Fig. 1.

## Discussion

The aim of this study was to examine the relationships between EF, MZ, and PTS symptoms in adult individuals exposed to earthquakes. Accordingly, I tested the moderating effect of CAMSQ SC, OC, and HMZ (other-self-discrepancy) on the relationship between EF and PCL. Our main hypothesis was that EF has a significant relationship with OC, SC, and HMZ, and that OC, SC, and HMZ may moderate the relationship between EF and PTS symptoms.

The results showed a negative correlation between SC and OC with EF, and a positive correlation between HMZ and EF. These findings are consistent with the results of correlational and neuroscientific studies supporting the relationship between impairments in EF and MZ impairments [25–27, 46–49]. Furthermore, both EF and MZ were positively and significantly correlated with PTS symptoms. Consistent with other research findings, our results show that impaired EF in traumatic stress situations is associated with increased PTS reactions [16, 17] Additionally, the results also indicated different relationships between SC, OC, HMZ, and PTS symptoms. Accordingly, while I found no significant relationship between SC and PTS symptoms, OC and HMZ had a significant positive relationship with PTS symptoms. These results are consistent with previous research findings that there is no significant relationship between self-mentalizing and psychopathological symptoms [50], while other-mentalizing and hypermentalizing have a positive and significant relationship with psychopathological symptoms [32, 50].

According to the results of the study on the moderating effects of SC, OC, and HMZ on the relationship between EF and PTS symptoms, SC and OC have no moderating effect on the relationship between EF and PTS symptoms, whereas HMZ positively moderates this relationship. The results are consistent with previous findings supporting the relationship between HMZ and psychopathology [32] and are consistent with research findings showing that impairment in EF is associated with impaired MZ, which in turn is associated with individuals displaying more psychological symptoms [27, 46, 47, 51].

Hypomentalizing (too little certainty about one's mental states) and hypermentalizing (too much certainty about others'mental states/Other-Self-Discrepancy) are related to psychopathological symptoms [32]. This theoretical framework provides an important basis for explaining the results within the context of container life and collective trauma. In the areas in which we collected data, the destructive effects of the earthquakes have rendered the cities uninhabitable, people started to live more collectively in container cities or rural areas. People, especially those living in container cities, have to struggle with the difficulties of this lifestyle in addition to the explicitly earthquake-related experiences. After such a major traumatic experience, survivors may develop a heightened fragility, making them more vulnerable to external factors [52]. In this context, I believe that participants in our study were aware of their mental state in relation to both the direct effects of earthquakes and their current life challenges. In addition, they may have focused more on external events and others'mental states rather than their internal processes, depending on the type of trauma. This external focus, shaped by collective trauma and shared grief, may foster heightened empathic sensitivity, leading to patterns of hypermentalizing.

Parada-Fernández et al. [53] found that showing more compassion and concern for others in the event of negative life events and witnessing others’ negative experiences may be associated with displaying increased discomfort and anxiety. Similarly, Schulte et al. [47] revealed that higher affective empathy was associated with social adjustment difficulties in individuals living in poverty. Similarly, when I consider OC has a positive correlation with empathy (especially emotional empathy) and emotion recognition skills [53], I suggest that too much certainty about others’ mental states (hypermentalizing/Other-Self-Discrepancy) in these circumstances may trigger further stress when combined with trauma memories and life difficulties. Because low EF may make it difficult for individuals to develop self-regulatory strategies to counteract their high empathetic anxiety [47]. In addition, in the case of EF impairments, individuals may experience problems with focusing on and planning processes that may help them adaptively process trauma-related symptoms. Thus, individuals may use maladaptive self-regulation strategies to cope with distress. This may be linked to the persistence and exacerbation of PTS symptoms [22]. Therefore, I suggest that impaired executive functioning, combined with emotional responses triggered by excessive certainty about others'mental states, may hinder self-regulation and contribute to the development or persistence of post-traumatic stress symptoms.

Consequently, in the earthquake-affected areas, the multiple losses and socioeconomic hardships caused by the earthquake resulted in multiple sources of stress for survivors. Temporary living conditions, such as container homes, can further increase stress levels among survivors. Therefore, survivors have experienced both acute traumatic events and long-term life challenges. In this context, the relationship I found between EF, HMZ, and PTS symptoms may reflect the psychological cost of being overly empathetic to others' suffering in a collective trauma environment. Based on these findings, I suggest that mentalizing tendencies, along with those characterized by weak EF, should be considered when assessing trauma responses, as these may hinder recovery and exacerbate distress.

### Theoretical and clinical implications

Our results provide support for the relationship between configurations of MZ (self- and other certainty) and psychological symptoms, proposed by Müller et al. [32], as well as the use of CAMSQ in this field. As far as I know, this study offers the first examination of the relationship between EF and MZ, on one hand, and PTS, on the other, in the context of earthquake trauma. I support the idea that MZ of others (or OC) and HMZ in chronic adversity are important variables in PTS symptoms. This corroborates studies showing that understanding others is associated with problems when it cannot be regulated in persistent adverse living conditions [47, 54]. Our study thus points to a relationship between two important neurocognitive components and PTS symptoms. In addition, the results show that it may be important to develop strategies to support both EF and MZ abilities in interventions to manage chronic life difficulties after disasters.

### Limitations of the study and suggestions for future research

Although our study makes important contributions to literature, it has important limitations. First, I had difficulties in comparing our research findings with the results of the previous research. Because there is an ongoing discussion in the research regarding the psychometric properties of the instruments most commonly used to assess MZ [55–58]. In addition, the CAMSQ [32, 38], which I used in our study, is new in this field, which introduced difficulties into our discussion of the results.

Second, since our research is based on a cross-sectional design, the results do not allow inferences about causality. Third, the study data were collected through self-report assessment tools. For example, the ADEXI, which I used for EF assessment, should be used as a complement to, rather than a substitute for, neuropsychological tests [33].

Fourth, I included the participants in the study through convenience sampling. The use of a convenience sampling method constitutes a limitation of the study, as it may restrict the generalizability of the findings to the broader earthquake-affected population. Therefore, further studies with more representative samples are needed to validate and extend these results.

Therefore, further validation of our results could be facilitated by an approach based on data diversity. In this respect, more empirical evidence could be obtained by using different assessment tools, such as interviews and performance-based tasks. In addition, the use of a longitudinal design could ameliorate the methodological limitations of the cross-sectional design on which our moderation analysis was based, which prevented the establishment of causal relationships between the study variables. Studies combining nomothetic and idiographic methods can also be conducted to address this data- and method-dependent problem and to confirm our research findings [59]. To this end, in addition to nomothetic assessments applied to all participants, an idiographic approach can be adopted for a select sample based on specific criteria. This approach aims to provide an in-depth examination of individual phenomena using qualitative (observation, interviews, or diaries) and quantitative data collection methods (self-reported scale, physiological, and neurocognitive measures). This approach, which combines qualitative and quantitative measurements, can provide a detailed and comprehensive understanding of individuals, including potential factors beyond the variables under investigation, and can reduce individual biases over time by documenting these individual responses as they occur. Furthermore, these intensive and repeated measurements deepen our understanding of individuals'responses to their context and changes within it. Thus, developing a more detailed understanding of the EF–MZ–PCL relationship may help address contextual conditions and guide the creation of individualized intervention strategies. Enhancing EF and fostering optimal mentalizing could support individuals in coping with prolonged post-disaster challenges, while targeted cognitive training and mentalization-based approaches may reduce vulnerability to chronic stress reactions.

## Conclusion

The results of this study show that impairment of EF and too much OC (MZ of others’ mental states) play a fundamental role in PTS reactions among adults who have experienced earthquake trauma and challenging post-earthquake challenging conditions. To corroborate the results of this study, it would be necessary to conduct studies that combine the nomothetic method with idiographic methods.

## Data Availability

No datasets were generated or analysed during the current study.

## Abbreviations

MZ: Mentalizing
EF: Executive Function
PTS: Post-Traumatic Stress
ADEXI: Adulthood Executive Functioning Inventory
CAMSQ: Certainty About Mental States Questionnaire
PCL: Post-Traumatic Stress Disorder Checklist
SC: Self-Certainty
OC: Other-Certainty
HMZ: Hypermentalizing (Other–Self Discrepancy)
PTSD: Post-Traumatic Stress Disorder
DSM-5: Diagnostic and Statistical Manual of Mental Disorders, Fifth Edition
CIs: Confidence intervals
